# Assessing the health-state utility values of rare disease-hemophilia B using EQ-5D-5L: a study based on the Chinese population

**DOI:** 10.1186/s13023-025-03894-y

**Published:** 2025-08-07

**Authors:** Chuchuan Wan, Haotao Li, Yulin Zhang, Qiqi Wang, Yiwen Huang, Tao Guan, Xiaoyu Xi

**Affiliations:** 1 The Research Center of National Drug Policy & Ecosystem, China Pharmaceutical University, 639 Longmian Avenue, Jiangning District, Nanjing, Jiangsu Province China; 2 School of International Pharmaceutical Business, China Pharmaceutical University, Nanjing, China; 3 Beijing Hemophilia Home Care Center, Beijing, China

**Keywords:** Health-state utility values, Hemophilia B, China, EQ-5D-5L

## Abstract

**Background:**

Obtaining health-state utility values (HSUVs) aids in making scientific decisions in patient health management, especially for rare disease patients. However, there is currently no research specifically measuring the HSUVs of Chinese hemophilia B patients. Therefore, this study aims to assess the HSUVs of hemophilia B patients in China and explore its potential influencing factors.

**Methods:**

The sociodemographic characteristics of patients were obtained from the Beijing Hemophilia Home Care Center (BHHCC) database. And the HSUVs were further obtained by reaching hemophilia B patients through an application of BHHCC and the Chinese version of the EQ-5D-5L. The beta regression model was used to explore the potential influencing factors of the HSUVs of patients.

**Results:**

A total of 167 male patients (hemophilia B is an X-chromosome recessive disorder and female patients are rare) were included in the study. The mean age, HSUV and EQ-VAS were 20.01 ± 15.83, 0.755 ± 0.291 and 71.7 ± 22.7, respectively. The ceiling effects was 29.24%, and patients were more likely to experience problems in Pain/discomfort (57.49%). Compared to self-completion, proxy may overestimate HSUVs of patients. Pain (*p* < 0.000), disability (*p* < 0.000), complications (*p* < 0.001), inhibitors (*p* < 0.01), drug usage (*p* < 0.001), and bleeds (*p* < 0.000) were significantly associated with HSUVs in Chinese hemophilia B patients.

**Conclusions:**

This study first assessed the HSUVs of Chinese hemophilia B patients, which provides support for further economic studies. Potential factors that affect the HSUVs of Chinese hemophilia B patients were also explored, which can provide a reference for developing health management measures. However, to enact more comprehensive and reliable disease management decisions, the effects of self-completion and proxy on the HSUVs of hemophilia B patients in China need to be further explored as well as the effects of specific factors.

**Supplementary Information:**

The online version contains supplementary material available at 10.1186/s13023-025-03894-y.

## Background

Hemophilia B is a rare X-chromosome recessive genetic disease that primarily affects males [1]. The incidence of hemophilia B in the world and China are approximately 3.8/100,000 [2] and 1/25,000 [1], respectively. Hemophilia B has been included in the Chinese Rare Disease Catalog [3]. The function abilities of clotting factor IX(FIX) are low in HB patients due to mutations in the gene encoding FI, leading to the dysfunction of coagulation [4, 5], like spontaneous bleeding, bleeding after minor trauma, severe bleeding after wound or surgery. Repeated bleeding for a long time will hinder the patient’s normal life, cause physical pain and even cause disability. These symptoms seriously affect the overall health of HB patients and bring a huge economic burden. Research shows that the average annual treatment cost for hemophilia patients reaches 129,365 euros in the UK and 196,117 euros in France [6]. And the annual treatment cost for hemophilia B patients in the United States is as high as 614,886 dollars [7]. Our past research results showed that the current treatment of hemophilia B patients in China is seriously insufficient, while the per capita annual treatment cost is still as high as 47,000 CNY and the resulting annual income loss per capita has reached 32,000 CNY [8]. It is foreseeable that the annual per capita treatment cost for Chinese hemophilia B patients may increase a lot in the future. 

In view of the heavy economic burden caused by hemophilia B and the limited health resources, it is significant to conduct health technology assessment of hemophilia B interventions. Cost-utility analysis is currently a widely recommended method for health technology assessment [9] by effectively quantifying health-related quality of life through quality-adjusted life years(QALYs) [10]. The quantification of QALYs requires the patient’s health-status utility values (HSUVs), which acquired from preference-based scales, such as the EQ-5D and SF-6D. At present, some studies have used the tools above to obtain HSUVs for hemophilia patients in China, the United States, the United Kingdom, France, Canada, etc. [6, 7, 11, 12] However, only three studies were based on Chinese hemophilia patients, each using SF-6D v2 [11], EQ-5D-3L [13] and EQ-5D-5L [14]. International studies showed that HSUVs might be different between hemophilia A and Hemophilia B [15, 16]. Unfortunately, no studies distinguish between the two and measure the HSUVs of Chinese hemophilia B patients.

Therefore, this study will measure the HSUVs of Chinese hemophilia B patients through EQ-5D-5L and explore possible related factors. The research aims to provide high-quality data on the health status of Chinese hemophilia B patients for health technology assessment work and scientific health decision-making.

## Methods

### Study design

We collected relevant data from the Beijing Hemophilia Home Care Center (BHHCC) database and use the Chinese version of EQ-5D-5L to collect patients’ HSUVs. More than 10,000 patients across the country have registered with the BHHCC and patients can use the “Hemophilia Home” application (APP) developed by the organization to record data, including patient sociodemographic information, disease status, and treatment information. For patients who are unable to complete information recording on their own, such as minors, the elderly, or other patients with limited mobility, their caregivers can fill in the information on their behalf.

This study further formulated the inclusion and exclusion criteria. Inclusion criteria: (1) Citizens of the People’s Republic of China; (2) Diagnosed with hemophilia B by a formal medical institution; (3) Voluntarily registered for the “Hemophilia Home” APP; (4) Active users of the “Hemophilia Home” APP and voluntarily participated in this study; Exclusion criteria: (1) Unconscious or unable to understand the questionnaire.

### Data collection

To ensure the availability, completeness and quality of research data, we completed data collection in five steps. The first step is to screen active hemophilia B patients in the database from September 1, 2020 to August 31, 2021. In the second step, active patients are invited to participate in the study. In the third step, the informed consent form and the Chinese version of the EQ-5D-5L questionnaire will be sent to the patients who are willing to participate through the APP. Patients who are unable to complete the information recording on their own will receive the proxy version of the EQ-5D-5L. The fourth step is to take back the questionnaires and acquire the corresponding patient’s sociodemographic information, disease information, treatment information, etc. from the platform of BHHCC. Reference was made to existing research designs when deciding the acquisition categories of data indicators [11, 13]. The sociodemographic information includes gender, age, BMI, marriage status, education, annual family income, etc. The disease information includes joint injury, disability status, suffering time after diagnosis, whether there are other diseases been diagnosed, pain frequency, etc. The treatment information includes treatment patterns, drug usage, and Coagulation factor supplementation condition, etc. Detailed indicators can be found in Table 1. The fifth step is to review and clean the case data. Two researchers will clean the data back-to-back that are filled too quick, contains logical errors or misses critical information. Disputed case data will be reviewed and decided by a third person.

### Measurements

EQ-5D-5L, as a preference-based general scale, can effectively obtain the HSUVs of subjects [17]. At present, the reliability and validity of EQ-5D-5L have been verified in China and are widely used [14, 18]. EQ-5D-5L consists of two parts, the description system and the EQ visual analog scale (EQ-VAS). The description system describes the subject’s health status from five levels (no problems, slight problems, moderate problems, severe problems, and unable to/extreme problems) of five dimensions (mobility, self-care, usual activities, pain/discomfort, and anxiety/depression), with a total of 5^5^ = 3125 health conditions. The EQ-VAS assesses subjects’ self-reported health along a straight line (0: worst health imaginable; 100: Best health imaginable). Each health status displayed by EQ-5D-5L can be converted into a HSUV through a value set constructed based on the local cultural background. This study used the Chinese value set [19].

### Data analysis

Since previous studies have shown that there may be differences in the results of EQ-5D by self-completion and proxy [20–22]. Thus, we not only conducted an overall analysis, but also conducted a subgroup analysis based on the filling method.

#### Descriptive statistics

Means, standard deviations, medians, 95% confidence interval (95%CI), frequencies, and percentages were used to describe the continuous variables like patient characteristics and outcomes. Frequency and percentage were for categorical variables. Distribution plots, skewness, and kurtosis were used to describe the distribution of patient HSUVs and EQ-VAS scores. We compared the differences in HSUVs and the responses in each dimension of EQ-5D-5L between the sample and the Chinese general population. And Kruskal–Wallis test and proportion test were used for significance test of differences.

#### Univariate analysis

In order to explore the factors related to patients’ HSUVs, we statistically analyzed patients’ HSUVs with different characteristics. Due to the skewed distribution of HSUVs, the Kruskal–Wallis Test, a non-parametric method, was employed to assess significance.

#### Multivariate analysis

Gevin that the HSUVs are skewed and truncated at 1, we used the beta model to construct a multiple linear regression to further analyze factors that may influence patient HSUVs. Beta model requires dependent variable value to be between 0–1 [23, 24]. However, the patients’ HSUVs ranges from −0.391 to 1, so we adjusted the patients’ HSUVs through the formula: [25] Adjusted HSUV = (original HSUV + 0.391)/1.391(when original HSUV is −0.391 or 1, we added or subtracted e^−12^ from the adjusted score to ensure Adjusted HSUV is between 0 and 1.) The adjusted HSUV meets the data requirements of the beta model in terms of both morphological distribution and value ranges. The selection of independent variables was based on the results of previous relevant studies and the results of univariate analysis in this study (factors with high correlation with HSUVs). In order to further reduce the multicollinearity problem between independent variables, we used spearman rank correlation to further screen independent variables. For two independent variables with a correlation coefficient higher than 0.8, we compared the correlation coefficients between the two variables with other independent variables and selected the highest two correlation coefficient for further comparison. The variable with a higher correlation coefficient will be deleted (correlation coefficient: very weak = 0–0.19; weak = 0.20–0.39; medium = 0.40–0.59; strong = 0.60–0.79; very strong = 0.80–1.00) [25, 26]. Spearman analysis results showed that age was highly correlated with the suffering time after diagnosis (see details attachment 1). Therefore, we abandoned age as an independent variable. The final included independent variables are seen in the multivariate analysis results. We tried to build a variety of models to evaluate the collinearity problem of the model through VIF and select the optimal model using AIC and BIC values. To test the stability of the model, we transformed variables types of BMI, annual family income, and suffering time after diagnosis or increase variables such as age.

All above analysis were performed on Microsoft® Excel 2021 and stata15.

## Results

### Sociodemographic characteristics

A total of 167 patients were included in the study, of which 70 were self-completion and 97 were proxy. The average age was respectively 20.01 ± 15.83, 35.94 ± 9.76, and 8.51 ± 6.78 years old (see attachment 2). All patients were male (hemophilia B is an X-chromosome recessive disease and female patients are rare), and the sample sources covered all regions in China. The dropout rate of patients was 16.77%, and more than 60% of the marriageable patients were not married. The annual family income of patients was mostly 30,000 CNY to 80,000 CNY (47.90%), and most of the patients participate in the Urban and Rural Resident Basic Medical Insurance (75.45%). In terms of disease, the average suffering time after diagnosis was 14.78 ± 13.29 years. Nearly half of the patients were in the severe stage of the disease (53.29%), with joint injury (56.89%) and complications (46.71%). At the same time, 60%−70% of patients suffered from acute/chronic pain in daily life, and the disability rate reached 37.13%. In terms of treatment, the proportion of patients with the three treatment patterns (on-demand, prophylaxis, and a combination of the two) was comparable. The proportion of patients with available drugs was nearly 95%, and hematogenic factors or recombinant factors were mostly used (82.04%), but only about 20% of patients had sufficient coagulation factor supplements. For details on patient characteristics, see Table 1.

**Table 1:** EQ-5D-5L scores of patients with different characteristics

Demographic characteristics	n = 167(%)	Mean ± SD	Median (Range)	95%CI	*p* values
Total	167(100.00%)	0.755 ± 0.291	0.848(− 0.158, 1.000)	(0.710, 0.799)	
EQ-VAS (Total)	167(100.00%)	71.719 ± 22.669	80.000(1.000, 100.000)	(68.255, 75.182)	
Gender					
Male	167(100.00%)	0.755 ± 0.291	0.848(− 0.158, 1.000)	(0.710, 0.799)	
Age (years, Mean ± SD)	20.01 ± 15.83				0.0001^***^
0–6	45(26.95%)	0.870 ± 0.233	1.000(0.031, 1.000)	(0.800, 0.939)	
7–12	35(20.96%)	0.952 ± 0.090	1.000(0.511, 1.000)	(0.921, 0.983)	
13–17	10(5.99%)	0.863 ± 0.285	0.976(0.074, 1.000)	(0.658, 1.067)	
18–45	67(40.12%)	0.597 ± 0.285	0.665(− 0.158, 1.000)	(0.527, 0.666)	
46–69	10(5.99%)	0.501 ± 0.314	0.467(− 0.005, 0.942)	(0.276, 0.725)	
BMI (Mean ± SD)	20.91 ± 4.94				0.1196
< 18.5	57(34.13%)	0.810 ± 0.252	0.942(0.074, 1.000)	(0.743, 0.877)	
[18.5,24)	67(40.12%)	0.769 ± 0.277	0.848(− 0.158, 1.000)	(0.701, 0.836)	
[24,28)	32(19.16%)	0.669 ± 0.322	0.758(− 0.064, 1.000)	(0.553, 0.785)	
≥ 28	11(6.59%)	0.636 ± 0.400	0.782(0.025, 1.000)	(0.367, 0.905)	
Marriage status					0.0001^***^
Below the legal marriage age	99(59.28%)	0.887 ± 0.206	0.951(0.031, 1.000)	(0.846, 0.928)	
Marriageable but unmarried	43(25.75%)	0.545 ± 0.293	0.621(− 0.158, 1.000)	(0.454, 0.635)	
Married	25(14.97%)	0.594 ± 0.287	0.677(− 0.064, 1.000)	(0.476, 0.712)	
Education					0.0001^***^
Preschool	45(26.95%)	0.870 ± 0.233	1.000(0.031, 1.000)	(0.800, 0.939)	
During primary school	30(17.96%)	0.955 ± 0.095	1.000(0.511, 1.000)	(0.919, 0.990)	
During secondary school	6(3.59%)	0.751 ± 0.291	0.895(0.334, 1.000)	(0.445, 1.057)	
Primary school	12(7.19%)	0.573 ± 0.372	0.742(− 0.064, 1.000)	(0.337, 0.809)	
Secondary school	35(20.96%)	0.569 ± 0.311	0.642(− 0.158, 1.000)	(0.462, 0.676)	
University	11(6.59%)	0.644 ± 0.257	0.690(− 0.005, 0.889)	(0.472, 0.817)	
Dropout (not enrolled in school)	28(16.77%)	0.710 ± 0.265	0.782(0.074, 1.000)	(0.608, 0.813)	
Area					0.0991^*^
Eastern	73(43.71%)	0.785 ± 0.304	0.942(0.064, 1.000)	(0.714, 0.856)	
Central	37(22.16%)	0.712 ± 0.274	0.747(0.158, 1.000)	(0.621, 0.804)	
Western	43(25.75%)	0.731 ± 0.293	0.831(0.023, 1.000)	(0.641, 0.821)	
Northeast	14(8.38%)	0.781 ± 0.260	0.826(0.130, 1.000)	(0.631, 0.931)	
Annual family income (10,000 CNY, Mean ± SD)	7.26 ± 9.23				0.0001^***^
(0,3)	46(27.54%)	0.610 ± 0.335	0.734(− 0.158, 1.000)	(0.510, 0.710)	
[3,8)	80(47.90%)	0.737 ± 0.279	0.831(− 0.064, 1.000)	(0.675, 0.799)	
[8,15)	20(11.98%)	0.928 ± 0.095	0.976(0.739, 1.000)	(0.884, 0.972)	
[15, + ∞)	21(12.57%)	0.973 ± 0.047	1.000(0.813, 1.000)	(0.952, 0.995)	
Medical insurance					0.2781
Urban and Rural Residents Basic Medical Insurance	126(75.45%)	0.728 ± 0.309	0.848(− 0.158, 1.000)	(0.674, 0.782)	
Urban Employee Basic Medical Insurance	19(11.38%)	0.720 ± 0.244	0.782(− 0.005, 1.000)	(0.603, 0.838)	
Unknow	22(13.17%)	0.937 ± 0.097	1.000(0.698, 1.000)	(0.894, 0.980)	
Assistance					0.0007^***^
No	56(33.53%)	0.816 ± 0.274	0.947(− 0.064, 1.000)	(0.743, 0.890)	
Yes	92(55.09%)	0.682 ± 0.304	0.781(− 0.158, 1.000)	(0.619, 0.745)	
Unknow	19(11.38%)	0.927 ± 0.101	1.000(0.698, 1.000)	(0.879, 0.976)	
Suffering time after diagnosis (years, Mean ± SD)	14.78 ± 13.29				0.0001^***^
(0,10)	85(50.90%)	0.866 ± 0.209	0.951(0.031, 1.000)	(0.821, 0.911)	
[10,20)	30(17.96%)	0.805 ± 0.287	0.938(0.023, 1.000)	(0.698, 0.912)	
[20,30)	21(12.57%)	0.571 ± 0.334	0.621(− 0.158, 1.000)	(0.419, 0.723)	
[30, + ∞)	31(18.56%)	0.526 ± 0.276	0.621(− 0.064, 0.951)	(0.425, 0.627)	
Disease stage					0.2272
Mild	3(1.80%)	0.737 ± 0.288	0.782(0.429, 1.000)	(0.021, 1.453)	
Moderate	73(43.71%)	0.804 ± 0.252	0.893(− 0.064, 1.000)	(0.745, 0.863)	
Severe	89(53.29%)	0.720 ± 0.318	0.831(− 0.158, 1.000)	(0.653, 0.786)	
Others	2(1.20%)	0.555 ± 0.094	0.555(0.488, 0.621)	(− 0.290, 1.399)	
Chronic pain frequency (past half year)					0.0001^***^
Never	40(23.95%)	0.958 ± 0.081	1.000(0.689, 1.000)	(0.932, 0.984)	
Rarely	68(40.72%)	0.825 ± 0.224	0.916(0.062, 1.000)	(0.771, 0.879)	
Sometimes	37(22.16%)	0.634 ± 0.246	0.734(− 0.005, 0.942)	(0.552, 0.716)	
Often	16(9.58%)	0.467 ± 0.326	0.455(− 0.064, 0.893)	(0.293, 0.641)	
Always	6(3.59%)	0.115 ± 0.250	0.036(− 0.158, 0.479)	(− 0.148, 0.378)	
Acute pain frequency (past half year)					0.0001^***^
Never	58(34.73%)	0.930 ± 0.123	1.000(0.424, 1.000)	(0.898, 0.962)	
Rarely	77(46.11%)	0.716 ± 0.264	0.782(0.062, 1.000)	(0.656, 0.776)	
Sometimes	26(15.57%)	0.545 ± 0.360	0.648(− 0.158, 1.000)	(0.400, 0.691)	
Often	6(3.59%)	0.465 ± 0.465	0.567(− 0.064, 0.893)	(− 0.024, 0.953)	
Joint injury					0.0001^***^
No	72(43.11%)	0.903 ± 0.191	1.000(0.031, 1.000)	(0.858, 0.948)	
Yes	95(56.89%)	0.642 ± 0.303	0.734(− 0.158, 1.000)	(0.580, 0.704)	
Disability level					0.0001^***^
No	105(62.87%)	0.883 ± 0.196	0.951(0.031, 1.000)	(0.845, 0.921)	
Grade 1	6(3.59%)	0.344 ± 0.378	0.216(0.023, 1.000)	(− 0.053, 0.741)	
Grade 2	27(16.17%)	0.466 ± 0.323	0.500(− 0.158, 1.000)	(0.338, 0.594)	
Grade 3	17(10.18%)	0.546 ± 0.207	0.511(0.216, 0.942)	(0.440, 0.652)	
Grade 4	12(7.19%)	0.781 ± 0.123	0.760(0.616, 1.000)	(0.703, 0.859)	
Complication					0.0001^***^
No	89(53.29%)	0.869 ± 0.212	0.951(0.031, 1.000)	(0.824, 0.914)	
Yes	78(46.71%)	0.624 ± 0.314	0.702(− 0.158, 1.000)	(0.554, 0.695)	
Infections					0.0009^***^
No	139(83.23%)	0.784 ± 0.275	0.897(− 0.005, 1.000)	(0.738, 0.830)	
Yes	28(16.77%)	0.611 ± 0.328	0.690(− 0.158, 1.000)	(0.484, 0.738)	
Inhibitor inside the body					0.0016^***^
No	111(66.47%)	0.808 ± 0.257	0.942(− 0.064, 1.000)	(0.760, 0.856)	
Yes	7(4.19%)	0.646 ± 0.477	0.897(− 0.158, 1.000)	(0.204, 1.087)	
Unknow	49(29.34%)	0.650 ± 0.305	0.735(− 0.064, 1.000)	(0.562, 0.737)	
Bleeds that may lead to disability or death (past half year)					0.0001^**^
No	136(81.4%)	0.799 ± 0.254	0.895(− 0.064, 1.000)	(0.756, 0.842)	
Yes	31(18.6%)	0.561 ± 0.360	0.621(− 0.158, 1.000)	(0.429, 0.693)	
Other disease					0.0001^***^
No	126(75.45%)	0.807 ± 0.268	0.942(− 0.158, 1.000)	(0.760, 0.854)	
Yes	32(19.16%)	0.607 ± 0.321	0.690(− 0.064, 0.951)	(0.492, 0.723)	
Unknow	9(5.39%)	0.549 ± 0.229	0.642(0.216, 0.827)	(0.372, 0.725)	
Treatment pattern					0.0001^***^
On-demand	58(34.73%)	0.652 ± 0.331	0.777(− 0.158, 1.000)	(0.565, 0.739)	
Prophylaxis	48(28.74%)	0.856 ± 0.220	0.951(0.062, 1.000)	(0.792, 0.920)	
Combination	61(36.53%)	0.773 ± 0.269	0.893(0.031, 1.000)	(0.704, 0.842)	
More treatment times than bleeding times					0.0005^***^
No	33(19.76%)	0.610 ± 0.331	0.690(− 0.158, 1.000)	(0.492, 0.727)	
Yes	134(80.24%)	0.791 ± 0.269	0.895(− 0.064, 1.000)	(0.744, 0.837)	
Drug usage					0.0079^***^
No	9(5.39%)	0.582 ± 0.371	0.698(0.031, 1.000)	(0.297, 0.868)	
Plasma	21(12.57%)	0.644 ± 0.303	0.734(− 0.158, 1.000)	(0.506, 0.782)	
Hematogenic factors/Recombinant factors	137(82.04%)	0.783 ± 0.277	0.897(− 0.064, 1.000)	(0.736, 0.830)	
Coagulation factor supplementation condition					0.0103^**^
Extremely insufficient	13(7.78%)	0.525 ± 0.382	0.500(− 0.158, 1.000)	(0.295, 0.756)	
Insufficient	87(52.10%)	0.735 ± 0.279	0.813(− 0.064, 1.000)	(0.676, 0.794)	
Sufficient	33(19.76%)	0.804 ± 0.296	0.942(0.025, 1.000)	(0.699, 0.909)	
Unknow	34(20.36%)	0.845 ± 0.226	0.947(0.269, 1.000)	(0.767, 0.924)	
Treatment receive condition (past two weeks)					0.0508^*^
No	89(53.30%)	0.787 ± 0.282	0.897(− 0.064, 1.000)	(0.728, 0.847)	
Yes	78(46.70%)	0.718 ± 0.297	0.796(− 0.158, 1.000)	(0.651, 0.785)	
Self-medication (past two weeks)					0.0849^*^
No	26(15.57%)	0.847 ± 0.201	0.942(0.302, 1.000)	(0.766, 0.929)	
Yes	141(84.43%)	0.738 ± 0.302	0.848(− 0.158, 1.000)	(0.687, 0.788)	
Be hospitalized (past year)					0.1314
No	122(73.05%)	0.770 ± 0.263	0.886(− 0.158, 1.000)	(0.718, 0.822)	
Yes	45(26.95%)	0.715 ± 0.043	0.813(0.031, 1.000)	(0.627, 0.802)	

*p<0.1, **p<0.05, ***p<0.001

### EQ-5D-5L dimensional response

**Fig. 1:**
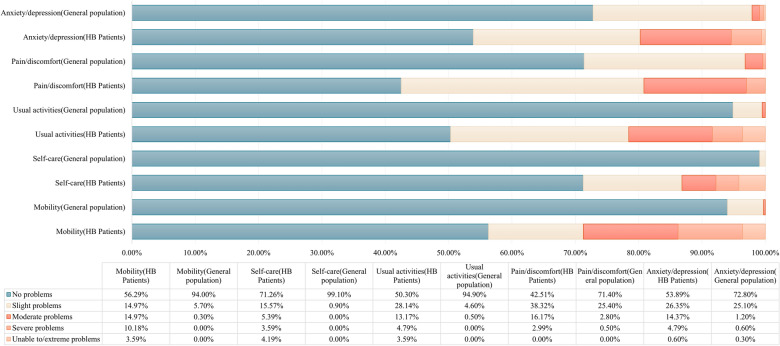
EQ-5D-5L dimensional response for all participants

A total of 50 patients (29.24%) reported no problems in all dimensions of EQ-5D-5L, including 2 patients (2.86%) in the self-completion group and 48 patients (50.00%) in the proxy group. The probability of patients having problems in all dimensions of EQ-5D-5L from high to low is Pain/discomfort (57.49%), Usual activities (49.70%), Anxiety/depression (46.11%), Mobility (43.71%), Self-care (28.74%), which were all significantly higher than the general Chinese population [27]. The rates of having problems in dimension of Pain/discomfort were the highest proportion in both groups, see Fig. 1. Patients in the self-completion group had significantly more problems than the general population in all dimensions of EQ-5D-5L. However, there was no significant difference between patients in the proxy and the general Chinese population in dimensions of Anxiety/depression, Pain/discomfort and Mobility. See attachment 3 for details.

### EQ-5D-5L score and distribution

**Fig. 2:**
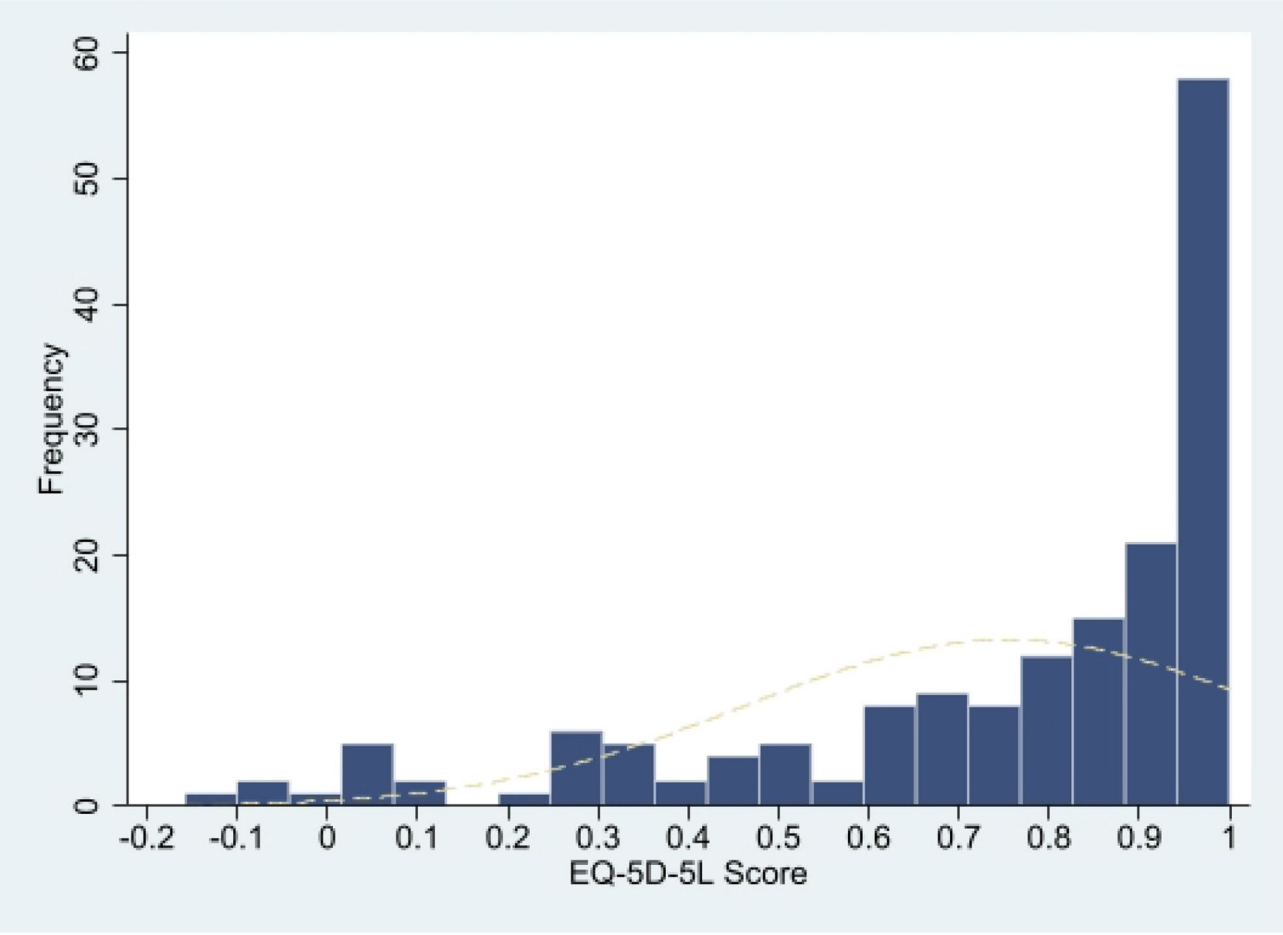
EQ-5D-5L Score for all participants

**Fig. 3:**
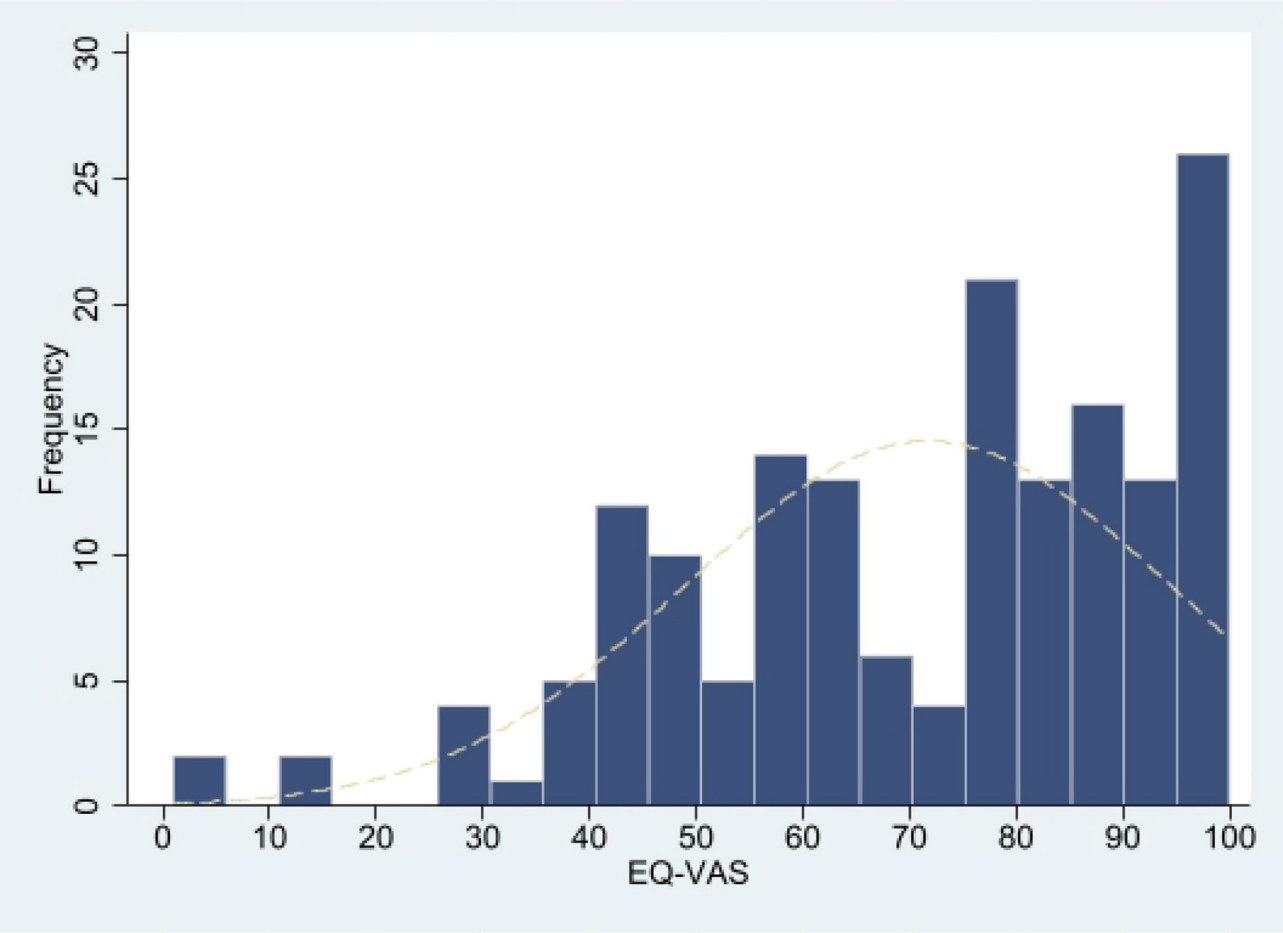
EQ-VAS for all participants

The HSUVs of patients was significantly lower than that of general Chinese population [27] (0.755 ± 0.291 VS 0.957 ± 0.069, *p* < 0.0000), showing a left-skewed distribution (skewness = −1.2985 < 0, *p* < 0.0000; kurtosis = 3.7936 > 3, *p* = 0.0558), see Fig. 2. The result of EQ-VAS was similar (71.7 ± 22.7 VS 86.0 ± 11.4, *p* < 0.0000; skewness = −0.6497 < 0, *p* = 0.0010; kurtosis = 2.8155 > 3, *p* = 0.7650), see Fig. 3. The HSUVs (self-completion, 0.570 ± 0.283; proxy, 0.888 ± 0.214) and EQ-VAS (self-completion, 58.043 ± 17.756; proxy, 81.588 ± 20.670) for self-completion group and the proxy group both were significantly lower than that of general Chinese population (*p* < 0.01). As for distribution, except that the EQ-VAS of the self-completion group is normally distributed, the other results were consistent with the main result. For details, see attachment 4 to 8.

### Univariable analysis results

HSUVs of patients were significantly lower in groups of older age (*p* = 0.0001), graduated with lower education level (*p* = 0.0001), lower annual family income (*p* = 0.0001), marriageable but unmarried (*p* = 0.0001), and not receiving assistance (*p* = 0.0007). In terms of disease status, the HSUVs of patients with longer suffering time after the diagnosis (*p* = 0.0001), higher frequency of acute/chronic pain (*p* = 0.0001), higher disability level (*p* = 0.0001), joint injury (*p* = 0.0001), complications (*p* = 0.0001), infection (*p* = 0.0009), inhibitors inside the body (*p* = 0.0016), bleeds that may lead to disability or death (*p* = 0.0001), and other diseases (*p* = 0.0001) were significantly lower. In terms of treatment, the HSUVs of patients were significantly higher in groups of employing prophylaxis treatment (*p* = 0.0001), higher treatments times than bleeding times (*p* = 0.0001), use of hematogenic factors/recombinant factors (*p* = 0.0079), and more sufficient coagulation factor (*p* = 0.0103). Details were shown in Table 1.

Compared with the results of all participants, in the self-completion group, only the patients with low annual family income (*p* = 0.0349), higher frequency of acute/chronic pain (*p* = 0.0001), higher disability level (*p* = 0.0001), joint injury (*p* = 0.0219), and bleeds that may lead to disability or death (*p* = 0.0372) had significantly lower HSUVs. Furthermore, the HSUVs of patients participating in the Urban and Rural Resident Basic Medical Insurance (*p* = 0.0168) were significantly lower. While in proxy group, the HSUVs of patients in groups of graduated with lower education level, higher disability level, joint injury, infection and more sufficient coagulation factor no longer showed significant differences, and the patients who had treatment in the past two weeks (*p* = 0.0364) or hospitalization in the past year (*p* = 0.0050) were significantly lower. Other characteristics are consistent with the results of all participant groups. For details on the results of the self-reported group and the proxy-reported group, see attachment 2.

### Multivariable analysis results

Beta regression analysis results showed (VIF = 1.92) that BMI (*p* < 0.05), disease stage (*p* < 0.001), disability level (*p* < 0.000), complications (*p* < 0.05), bleeds that may lead to disability or death (*p* < 0.000), acute/chronic pain frequency and score (*p* < 0.000), drug usages (*p* < 0.001), coagulation factor satisfaction status (*p* < 0.05), self-medication (*p* < 0.01) were significantly related to patient’s HSUV. Factors such as joint injury (*p* < 0.1), inhibitors inside the body (*p* < 0.1) and other diseases (*p* < 0.1) may be related to the patient’s HSUV. See Table 2 for details.

**Table 2:** Multivariate analysis results for all participants

Utility		Coefficient	Std. err	z	*p* > z	[95% conf. interval]
BMI						
< 18.5	Ref					
[18.5,24)	0.490	0.214	2.290	0.0220^**^	0.071	0.909
[24,28)	0.471	0.240	1.960	0.0500^**^	0.001	0.942
≥ 28	− 0.017	0.317	− 0.050	0.9580	− 0.638	0.605
Marriage						
Below the legal marriage age	Ref					
Marriageable but unmarried	− 0.256	0.384	− 0.670	0.5050	− 1.009	0.497
Married	− 0.288	0.437	− 0.660	0.5090	− 1.144	0.568
Education						
Preschool	Ref					
During primary school	0.433	0.291	1.490	0.1370	− 0.138	1.004
During secondary school	− 0.293	0.466	− 0.630	0.5290	− 1.206	0.620
Primary school	0.317	0.516	0.610	0.5390	− 0.695	1.329
Secondary school	− 0.415	0.405	− 1.020	0.3060	− 1.209	0.379
University	− 0.442	0.495	− 0.890	0.3710	− 1.412	0.528
Dropout (not enrolled in school)	− 0.099	0.355	− 0.280	0.7800	− 0.795	0.597
Area						
Eastern	Ref					
Central	0.059	0.227	0.260	0.7940	− 0.386	0.505
Western	− 0.132	0.231	− 0.570	0.5670	− 0.585	0.321
Northeast	0.458	0.288	1.590	0.1120	− 0.107	1.023
Annual family income	− 0.007	0.012	− 0.560	0.5790	− 0.030	0.017
Medical insurance						
Urban and Rural Residents Basic Medical Insurance	Ref					
Urban Employee Basic Medical Insurance	0.243	0.253	0.960	0.3350	− 0.252	0.739
Unknow	2.301	0.846	2.720	0.0070^***^	0.643	3.959
Assistance						
No	Ref					
Yes	0.080	0.187	0.430	0.6680	− 0.286	0.446
Unknow	− 2.443	0.869	− 2.810	0.0050^***^	− 4.145	− 0.741
Suffering time after diagnosis	− 0.006	0.010	− 0.540	0.5890	− 0.026	0.015
Disease stage						
Mild	Ref					
Moderate	1.733	0.583	2.970	0.0030^***^	0.591	2.875
Severe	1.767	0.563	3.140	0.0020^***^	0.664	2.870
Others	2.640	0.766	3.450	0.0010^***^	1.140	4.141
Joint injury						
No	Ref					
Yes	0.429	0.255	1.680	0.0930^*^	− 0.072	0.929
Disability level						
No	Ref					
Grade 1	− 1.548	0.412	− 3.760	0.0000^***^	− 2.356	− 0.740
Grade 2	− 1.779	0.322	− 5.520	0.0000^***^	− 2.411	− 1.148
Grade 3	− 0.931	0.293	− 3.180	0.0010^***^	− 1.505	− 0.356
Grade 4	− 1.298	0.349	− 3.720	0.0000^***^	− 1.981	− 0.615
Complication						
No	Ref					
Yes	− 0.520	0.237	− 2.200	0.0280^**^	− 0.985	− 0.056
Infections						
No	Ref					
Yes	− 0.041	0.245	− 0.170	0.8660	− 0.521	0.438
Inhibitor in the body						
No	Ref					
Yes	− 0.771	0.437	− 1.760	0.0780^*^	− 1.628	0.086
Unknow	− 0.264	0.199	− 1.320	0.1860	− 0.654	0.127
Other disease						
No	Ref					
Yes	− 0.368	0.217	− 1.690	0.0900^*^	− 0.793	0.058
Unknow	− 0.667	0.373	− 1.790	0.0740^*^	− 1.399	0.065
Bleeds number (past half year)	− 0.003	0.007	− 0.400	0.6860	− 0.018	0.012
Bleeds that may lead to disability or death (past half year)						
No	Ref					
Yes	− 0.887	0.246	− 3.600	0.0000^***^	− 1.370	− 0.404
Chronic pain score (past half year)	− 0.178	0.054	− 3.330	0.0010^***^	− 0.283	− 0.073
Acute pain score (past half year)	0.079	0.048	1.640	0.1010	− 0.015	0.173
Chronic pain frequency (past half year)						
Never	Ref					
Rarely	− 0.057	0.293	− 0.190	0.8460	− 0.632	0.517
Sometimes	− 0.874	0.369	− 2.370	0.0180^**^	− 1.597	− 0.150
Often	− 1.020	0.454	− 2.240	0.0250^**^	− 1.911	− 0.129
Always	− 1.306	0.523	− 2.500	0.0130^**^	− 2.332	− 0.281
Acute pain frequency (past half year)						
Never	Ref					
Rarely	− 1.049	0.271	− 3.870	0.0000^***^	− 1.580	− 0.517
Sometimes	− 0.636	0.365	− 1.740	0.0820^*^	− 1.352	0.080
Often	− 0.481	0.621	− 0.770	0.4390	− 1.699	0.737
More treatment times than bleeding times						
No	Ref					
Yes	0.013	0.240	0.050	0.9570	− 0.457	0.483
Treatment pattern						
On-demand	Ref					
Prophylaxis	− 0.252	0.300	− 0.840	0.4020	− 0.839	0.336
Combination	0.349	0.247	1.410	0.1570	− 0.135	0.832
Drug usage						
No	Ref					
Plasma	1.539	0.495	3.110	0.0020^***^	0.568	2.510
Hematogenic factors/Recombinant factors	1.377	0.430	3.200	0.0010^***^	0.533	2.220
Coagulation factor supplementation condition						
Extremely insufficient	Ref					
Insufficient	− 0.610	0.341	− 1.790	0.0740^*^	− 1.279	0.059
Sufficient	− 0.778	0.389	− 2.000	0.0460^**^	− 1.541	− 0.015
Unknow	− 0.492	0.378	− 1.300	0.1930	− 1.232	0.248
Treatment receive condition (past two weeks)						
No	Ref					
Yes	− 0.078	0.166	− 0.470	0.6380	− 0.403	0.247
Self-medication (past two weeks)						
No	Ref					
Yes	− 0.729	0.249	− 2.930	0.0030^***^	− 1.217	− 0.241
Be hospitalized (past year)						
No	Ref					
Yes	− 0.202	0.205	− 0.990	0.3240	− 0.605	0.200
Cons	2.551	0.978	2.610	0.0090^***^	0.635	4.467
scale						
Cons	2.314	0.131	17.610		2.056	2.571
Beta regression	Number of obs = 167					
	LR chi2(56) = 273.55					
	Prob > chi2 = 0.0000					
Link function:	g(u) = log(u/(1 − u)) [Logit]					
Slink function:	g(u) = log(u) [Log]					
Log likelihood =	514.13358					
Model	N	ll(null)	ll(model)	df	AIC	BIC
	167	377.3571	514.1336	58	− 912.2672	− 731.4235
VIF	1.92					

*p<0.1, **p<0.05, ***p<0.001

In the self-reported group (VIF = 2.14), BMI (*p* < 0.000), area (*p* < 0.000), annual family income (*p* < 0.000), medical insurance (*p* < 0.000), assistance (*p* < 0.000), suffering time after diagnosis (*p* < 0.001), disability level (*p* < 0.000), infection (*p* < 0.01), other diseases (*p* < 0.01), number of bleeding (*p* < 0.000), acute/chronic pain frequency and score (*p* < 0.000), treatment pattern (*p* < 0.000), self-medication (*p* < 0.000) were significantly related to the patient’s HSUV. Factors such as complications (*p* < 0.1) and inhibitors inside the body (*p* < 0.1) may be related to the patient’s HSUV. In the proxy group (VIF = 2.23), educational (*p* < 0.01), disease stage (*p* < 0.001), joint injury (*p* < 0.05), disability level (*p* < 0.05), complications (*p* < 0.001), inhibitors inside the body(*p* < 0.01), other diseases (*p* < 0.000), bleeds that may lead to disability or death (*p* < 0.05), an acute/chronic pain frequency and score (*p* < 0.05), drug usage (*p* < 0.01) were significantly related to patients’ HSUV. Factors such as area (*p* < 0.1), suffering time after diagnosis (*p* < 0.1), and treatment receive condition (past two weeks) (*p* < 0.1) might be related to patients’ HSUV. Details were shown in attachment 9.

Model robustness test results (See attachment 10 for details) showed that the regression model and results have good robustness.

## Discussion

This study first measure HSUVs of Chinese hemophilia B patient. As a rare disease, the number of patients with hemophilia B is relatively small. We expanded the sample size as much as possible and our participants come from all regions of the country. The proportion is similar to that of the Seventh National Population Census Bulletin issued [28] by the Chinese government (p > 0.1). In addition, the average sample age is similar to that of our previously published large sample study [8] (*p* > 0.1). Therefore, the results of the study have good representativeness in the Chinese hemophilia B patients. In this study, we measured the HSUVs of Chinese hemophilia B patients with different characteristics and analyzed the potential influencing factors, which can provide a reference for the health management of hemophilia B patients in China or other countries with similar national conditions. It also provides data for health economic evaluation, so as to provide decision-making information for the efficient use of medical resources.

Our results showed that the average HSUVs of Chinese hemophilia B patients was significantly lower than that of the general Chinese population [27] (0.957, *p* < 0.01), which was similar to the results of other studies [6, 11, 13, 29]. Compared with the existing research in China, which employed tools including SF-6D [11, 14], EQ-5D-3L [13] and EQ-5D-5L [14] and do not distinguish the hemophilia type, the HSUVs of the whole sample of patients in our study was slightly higher and the patients in the self-completion group were relatively lower. These results also apply when compared to the HSUVs of hemophilia B patients in the UK and France (0.700, measured using the EQ-5D-5L) [6]. It is noteworthy that although there was one study [13] in China reporting the HSUVs of hemophilia B patients, the sample size was only 16. Therefore, it might not effectively provide the HSUVs of Chinese hemophilia B patients and was well compensated by our study.

The results of our study showed that the HSUVs of mild patients were lower than those of moderate and severe patients (0.737, 0.804, 0.720), which was consistent with a Canadian study [30] of hemophilia A patients. This may due to the low number of mild patients included. As for the difference in HSUV between moderate and severe patients, our results and those of previous studies [6, 11, 31] showed that moderate patients were higher than severe patients. Our results for the all participants group are moderate compared to the results from other studies [6, 7, 32] using EQ-5D, but higher than those measured using TTO [31] or SF-6D [11]. While the results of the self-completion group were significantly lower than those of the above studies. In the choice of treatment pattern, patients treated with prophylaxis had higher HSUVs (on demand, prophylaxis, combination: 0.652, 0.856,0.773), which differs from the results of the two European study [6, 32] but be supported by the results of two studies based on Chinese population [11, 13] and one study based on European and American countries [12]. Compared with the results of the above studies, the results of the all participants group in our study were moderate, but lower for self-completion group. It is worth noting that almost all the studies mentioned above did not distinguish between hemophilia A and B patients, and there is also controversy regarding whether the HSUVs of hemophilia A and B patients is consistent [15, 16, 33]. However, by comparing the results of our study and the results of existing studies, HSUVs appears to be lower in hemophilia B patients. This may be due to different measurement tools and cultural backgrounds [34–36], which needs to be explored further in a prescriptive way.

About the responses of each dimension of EQ-5D-5L, the results of the all participants group in our study were similar to those of the Chinese general population [27] and other studies [14, 32]. Most problems occurred in the dimension of Pain/discomfort and least problems occurred in the dimension of self-care. However, Chinese hemophiliacs [14] are more prone to problems than foreign populations [32]. By comparison, we found that differences exist between proxy and self-completion. Among them, the arising order of problems in each dimension of the proxy group was completely consistent with that of the Chinese general population. Patients in the self-completion group reported more problems with mobility. Another notable issue is that 29.24% of patients report no problems across all dimensions of the EQ-5D-5L, suggesting that the EQ-5D-5L still exhibits a substantial ceiling effect among hemophilia B patients, especially in proxy group. While this phenomenon aligns with current researchers’ understanding of the EQ-5D-5L, it is essential to acknowledge its limitations in distinguishing patients with “very good” and “excellent” health. This may obscure variations in quality of life among healthier patients. Regarding the issues mentioned above, further research is needed to discuss the differences between the proxy and self-completion.

The HSUVs of patients decreases with age, which has been confirmed [37]. In addition, we found that the HSUVs of patients increase with suffering time after diagnosis, and elderly patients have significantly lower HSUV than younger patients. This may be due to the fact that patients suffer from more severe disease as they get older, such as more serious joint injury, and elderly patients have relatively less access to medical resources. Other findings [33, 38] appeared to explain this inference, but are subject to further confirmation. As for the phenomenon that the HSUVs of patients who have not reached the age of marriage or are still in school are higher, we believe the difference may be caused by the reporting method [11, 38]. However, in the proxy group, we found the HSUVs of marriageable but unmarried patients were significantly lower than those of patients under marriageable age. We suspect this may be caused by the caregiver’s concern about the patient’s future marital status and ability to shoulder family responsibilities. In addition, the results show that the higher the annual household income, the higher the HSUVs of patients, which is consistent with the existing research results [11]. The underlying reason may be that patients with higher incomes have better access to treatment and a reduced impact on their lives. This may corroborate our previous speculation that caregivers are concerned about the ability of patients to shoulder family responsibilities.

In terms of disease status, our findings were similar to those of previous studies. The patients with higher disability level [39], more joint injury [6, 40], more severe complications [11], more pain [41] had lower HSUVs. Previous studies have shown that bleeding may be not directly related to the HSUVs of patients and bleeding may indirectly affect the HSUVs of patients by affecting the joints [42]. But we found that patients with bleeding that may lead to disability or death had significantly reduced HSUVs. In addition, our study showed that infection may reduce the health benefits of patients, which is supported by studies in the Netherlands [43] and Sweden [29]. But an American study [42] has come to the opposite conclusion. The same problem arises with the relationship of the inhibitor inside the body and the HSUVs of patients. Our results suggested that inhibitors may reduce patient HSUVs, which was supported by the US study [42]. But another study [12] had the opposite conclusion. We believe that the instability of the results is caused by the small number of patients with infection and inhibitors in these studies. And larger samples are needed.

As for the influence of disease treatment on HSUV, in addition to the treatment way we mentioned above, we found that the HSUVs of patients using hematogenic factors/recombinant factors was higher than that of patients using plasma. This may be caused by the use of plasma increases the risk of infection, which in turn reduces the HSUVs of patients. Regarding the impact of coagulation factors supplementation condition, our results are consistent with another study findings [44] in China.

Comprehensively considering various analysis results, we believe that the factors such as degree and frequency of pain, disability level, complications, inhibitors inside the body, drug usage, and bleeds that may lead to disability or death may tend to affect the health of patients more.

We have to admit that there are some limitations in this study. First of all, we can only identify factors that may affect the HSUVs of patients instead of the causal relationship based on cross-sectional data. To explore the causal relationships, future investigations would require longitudinal data with temporal granularity for more robust analysis. Secondly, the questionnaire was voluntarily completed by patients through the app online, which may accidentally exclude those patients who were not accessible to the internet, lacking motivation, with lower economic status and severe health conditions. Although we attempted to obtain information from these patients through proxies, the occurrence of this issue could not be entirely prevented. At the same time, there is a lack of investigators to help patients understand the questionnaire. Thirdly, we expanded the sample size collection as much as possible, but the number of patients in our study with some characteristics was still low, such as mild patients, patients with inhibitors inside the body, etc. These may compromise the generalizability of the results. Fourthly, to expand the sample size, we included questionnaires partially completed by patient caregivers, which introduced proxy and self-completion biases. To address this limitation, we conducted subgroup analyses that identified substantial discrepancies between the two reporting modes, potentially affecting the representativeness of the overall findings. Regarding outcomes from different reporting methods, we suggest that future studies with larger cohorts may be required to delineate these variations. Fifth, we used the beta model. To meet the data requirements of the beta model, HSUV was adjusted using the formula [25] that had been validated and applied in existing research. But its application inevitably introduces some bias into the results. Finally, the Chinese value set for EQ-5D-Y-3L was not yet established during the data collection of this study, hence we did not use EQ-5D-Y-3L to measure the HSUVs of children. It is necessary for future studies to address this deficiency.

## Conclusion

The HSUVs of Chinese hemophilia B patients with different characteristics are provided, which is lower than that of the Chinese general population and the hemophilia patients in other countries, and its potential influencing factors are analyzed. The results can support the further economic studies and the formulation of health management measures for hemophilia B patients in China or other countries with similar national conditions. In addition, more rigorous studies are needed to explore the factors that may affect the HSUVs of hemophilia B patients and support disease management decisions. The effects of self-completion and proxy on the HSUVs of hemophilia B patients in China are equally worth exploring.

## Supplementary Information

Additional file 1.

## Data Availability

The datasets generated and/or analyzed during the current study are not publicly available due to being utilized for other studies but are available from the corresponding author on reasonable request.

## Abbreviations

QALYs: Quality-Adjusted Life Years
HSUVs: Health-State Utility Values
BHHCC: Beijing Hemophilia Home Care Center
EQ-VAS: EQ Visual Analog Scale
